# Supplementary material for: Patterns of connectome variability in autism across five functional activation tasks: findings from the LEAP project
Source: Mol Autism. 2022 Dec 27;13:53. doi: 10.1186/s13229-022-00529-y (PMC9793684; doi:10.1186/s13229-022-00529-y)
Supplement: Supplementary file 1 — Additional file 1: Scanning parameters, task description, subsample characterisations and additional figures. [file 13229_2022_529_MOESM1_ESM.pdf]

Patterns of connectome variability in autism across five functional activation tasks. Findings from the LEAP project

Supplementary information

fMRI scanning Parameters

Structural images were obtained using a 5.5-minute MPRAGE sequence (TR=2300ms, TE=2.93ms, T1=900ms, voxels size=1.1x1.1x1.2mm, flip angle=9°, matrix size=256x256, FOV=270mm, 176 slices). The rsfMRI scan was acquired using a multi-echo planar imaging (ME-EPI) sequence; TR=2300ms, TE 12ms, 31ms, and 48ms (slight variations are present across centres), flip angle=80°, matrix size=64x64, in-plane resolution=3.8mm, FOV=240mm, 33 axial slices, slice thickness/gap=3.8mm/0.4mm, volumes=200 (UMCU), 215 (KCL, CIMH), or 265 (RUNMC, UCAM).

fMRI task brief descriptions

Hariri: participants view a trio of faces and are asked to select one of two test faces that expresses the same emotion as the target face.

Flanker: participants press a button corresponding to the direction of an arrow target. The target is flanked by stimuli that point either in the same (congruent) or opposite (incongruent) direction, or by neutral flankers.

Social reward: An arrow cue indicates to the participants whether a given trial provides an opportunity for a win. Participants are then asked to make a fast button press in response to a target. Succesful eligible trials are rewarded with a happy face stimulus.

Social reward: An arrow cue indicates to the participants whether a given trial provides an opportunity for a win. Participants are then asked to make a fast button press in response to a target. Succesful eligible trials are rewarded with a monetary stimulus.

Theory of mind: participants are presented with short videos in which two triangles either move randomly, in a goal-directed manner, or in a goal directed manner that involves that manipulation of thoughts and feelings of the other triangle. Participants are asked to categorize the videos with one of these three labels.

Subsample characterizations per task

Table S1 (hariri)

variable	ASD mean(std) [min-max]	TD mean(std) [min-max]	post hoc
**age**	18.22(5.74) [7.58-30.0]	17.55(5.49) [6.89-30.0]	ns
**IQ**	106.93(16.29) [63.18-148.0]	108.03(11.99) [76.82-134.0]	ns
**SRS social responsiveness**	68.15(11.87) [43.0-95.0]	54.75(10.89) [37.0-67.0]	ASD>TD
**RBS repetitive behavior**	13.83(13.21) [0.0-90.0]	3.6(3.87) [0.0-23.0]	ASD>TD
**SSP sensory profile**	146.28(24.03) [53.0-190.0]	166.04(15.55) [138.0-190.0]	ASD<TD
**ADHD hyperimpulsivity**	2.15(2.64) [0.0-9.0]	0.52(1.05) [0.0-6.0]	ASD>TD
**ADHD inattentiveness**	3.79(2.86) [0.0-9.0]	1.86(1.69) [0.0-7.0]	ASD>TD
**VL adaptive functioning**	73.19(12.25) [20.0-117.0]	79.39(11.73) [73.0-126.0]	ASD<TD

Table S2 (flanker)

variable	ASD mean(std) [min-max]	TD mean(std) [min-max]	post hoc
**age**	19.16(4.77) [12.07-30.0]	18.75(4.61) [11.18-30.0]	ns
**IQ**	104.86(16.54) [63.18-148.0]	108.02(11.58) [76.82-133.0]	ns
**SRS social responsiveness**	68.19(11.7) [43.0-95.0]	55.6(10.83) [37.0-67.0]	ASD>TD
**RBS repetitive behavior**	14.18(13.43) [0.0-90.0]	3.78(3.81) [0.0-23.0]	ASD>TD
**SSP sensory profile**	147.78(23.29) [53.0-190.0]	165.44(15.63) [146.0-190.0]	ASD<TD
**ADHD hyperimpulsivity**	2.0(2.42) [0.0-9.0]	0.5(0.96) [0.0-6.0]	ASD>TD
**ADHD inattentiveness**	3.81(3.03) [0.0-9.0]	2.07(1.68) [0.0-7.0]	ASD>TD
**VL adaptive functioning**	71.67(11.46) [20.0-101.0]	75.06(3.84) [73.0-114.0]	ASD<TD

Table S3 (social reward)

variable	ASD mean(std) [min-max]	TD mean(std) [min-max]	post hoc
**age**	16.95(5.51) [7.48-30.0]	16.71(5.61) [6.89-30.0]	ns
**IQ**	101.06(18.62) [59.0-148.0]	106.8(13.82) [70.41-142.0]	ASD<TD
**SRS social responsiveness**	69.54(11.43) [43.0-95.0]	54.76(10.84) [37.0-73.0]	ASD>TD
**RBS repetitive behavior**	15.27(13.48) [0.0-90.0]	3.47(3.87) [0.0-23.0]	ASD>TD
**SSP sensory profile**	143.88(23.1) [53.0-190.0]	166.47(15.26) [138.0-190.0]	ASD<TD
**ADHD hyperimpulsivity**	2.38(2.59) [0.0-9.0]	0.59(1.23) [0.0-7.0]	ASD>TD
**ADHD inattentiveness**	4.16(3.0) [0.0-9.0]	1.88(1.71) [0.0-7.0]	ASD>TD
**VL adaptive functioning**	72.62(12.11) [20.0-117.0]	81.58(13.69) [66.0-127.0]	ASD<TD

Table S4 (monetary reward)

variable	ASD mean(std) [min-max]	TD mean(std) [min-max]	post hoc
age	16.99(5.49) [7.48-30.0]	16.71(5.7) [6.89-30.0]	ns
IQ	100.9(18.56) [59.0-148.0]	106.92(13.95) [70.41-142.0]	ASD<TD
SRS social responsiveness	69.59(11.4) [43.0-95.0]	54.75(10.74) [37.0-71.0]	ASD>TD
RBS repetitive behavior	15.14(13.36) [0.0-90.0]	3.51(3.86) [0.0-23.0]	ASD>TD
SSP sensory profile	144.18(22.73) [53.0-190.0]	166.28(15.18) [138.0-190.0]	ASD<TD
ADHD hyperimpulsivity	2.38(2.59) [0.0-9.0]	0.54(1.17) [0.0-7.0]	ASD>TD
ADHD inattentiveness	4.13(2.97) [0.0-9.0]	1.81(1.64) [0.0-6.0]	ASD>TD
VL adaptive functioning	72.67(12.03) [20.0-117.0]	81.72(13.77) [66.0-127.0]	ASD<TD

Table S5 (theory of mind)

variable	ASD mean(std) [min-max]	TD mean(std) [min-max]	post hoc
age	16.97(5.75) [7.48-30.0]	16.64(5.47) [6.89-30.0]	ns
IQ	105.04(16.37) [63.18-148.0]	108.22(12.11) [74.0-134.0]	ASD<TD
SRS social responsiveness	69.33(11.57) [43.0-95.0]	54.31(10.87) [37.0-73.0]	ASD>TD
RBS repetitive behavior	14.65(12.7) [0.0-90.0]	3.33(3.82) [0.0-23.0]	ASD>TD
SSP sensory profile	144.76(23.56) [53.0-190.0]	166.94(15.41) [138.0-190.0]	ASD<TD
ADHD hyperimpulsivity	2.41(2.67) [0.0-9.0]	0.51(1.05) [0.0-6.0]	ASD>TD
ADHD inattentiveness	4.11(2.98) [0.0-9.0]	1.77(1.67) [0.0-7.0]	ASD>TD
VL adaptive functioning	73.69(11.67) [20.0-117.0]	81.29(13.05) [73.0-126.0]	ASD<TD

Full statistical values for figure 1

Table T1

	HARIRI	FLANKER	REWARD_M	REWARD_S	TOM
T-STATISTIC	-6,674	-3,907	-2,702	-2,805	-4,668
P-VALUE	1.592e-10	0,000117	0,00716	0,00530	4,338e-6
COHEN'S D	0,61	0,33	0,19	0,2	0,36

Supplementary figures

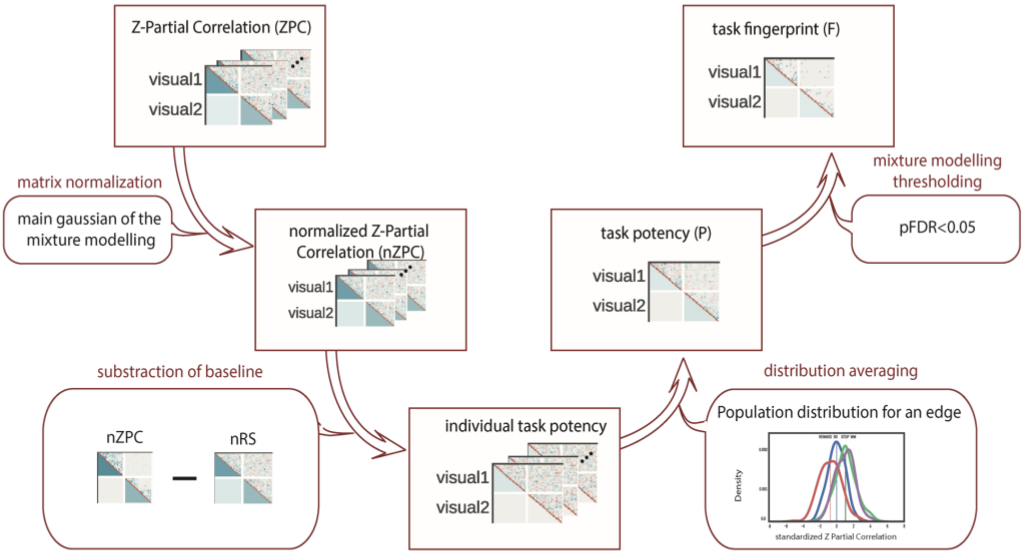

Figure supp 1. Visual analytical pipeline for task-potency

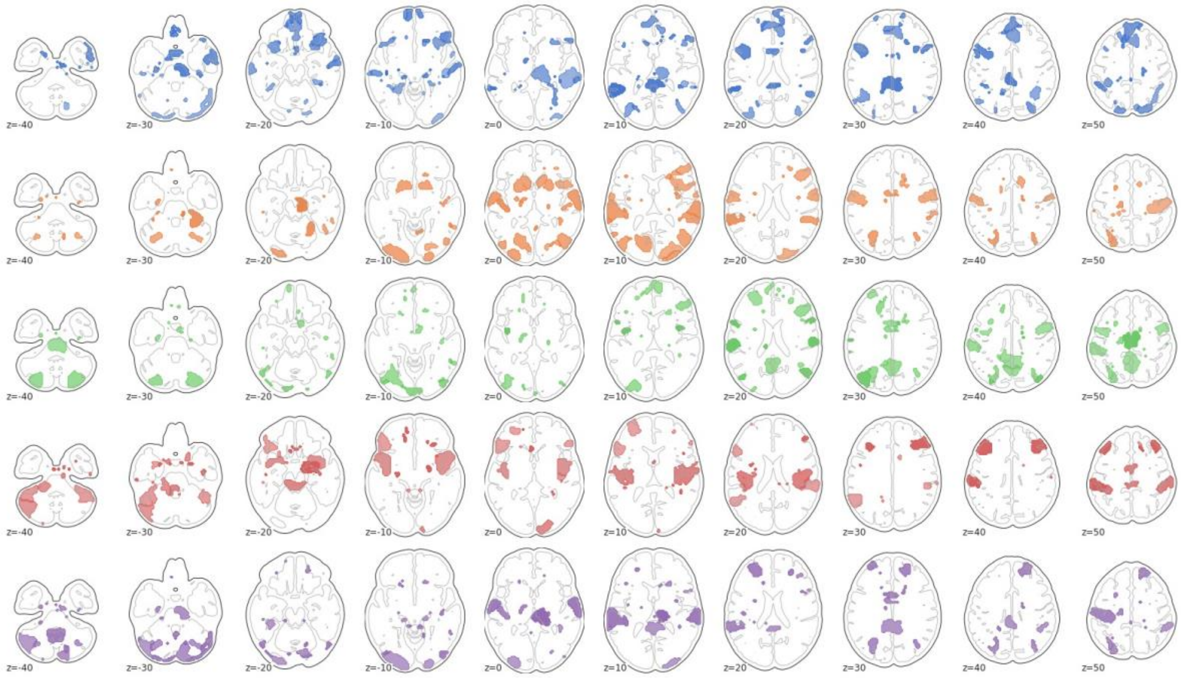

Figure supp 2. The top 10% brain regions with the greatest difference between normative modeling atypicality scores and CCA loadings in autism as summed over edges. From top to bottom: Hariri, Flanker, social reward, nonsocial reward, and theory of mind.

Figures 3A-E

Regression plots for each of the five tasks showing the relationships between participant scanner Framewise Displacement (FWD) and mean atypicality. Because our metric involves data from task- as well as resting state fMRI, we quantify participant FWD as the average of their task- and resting state fMRI FWD values. Hariri ($p = 0.106$), flanker ($p = 0.095$), social reward ($p = 0.819$), monetary reward ($p = 0.463$), theory of mind ($p = 0.822$). Main regression line and 95% confidence interval bounds are shown in the figures.

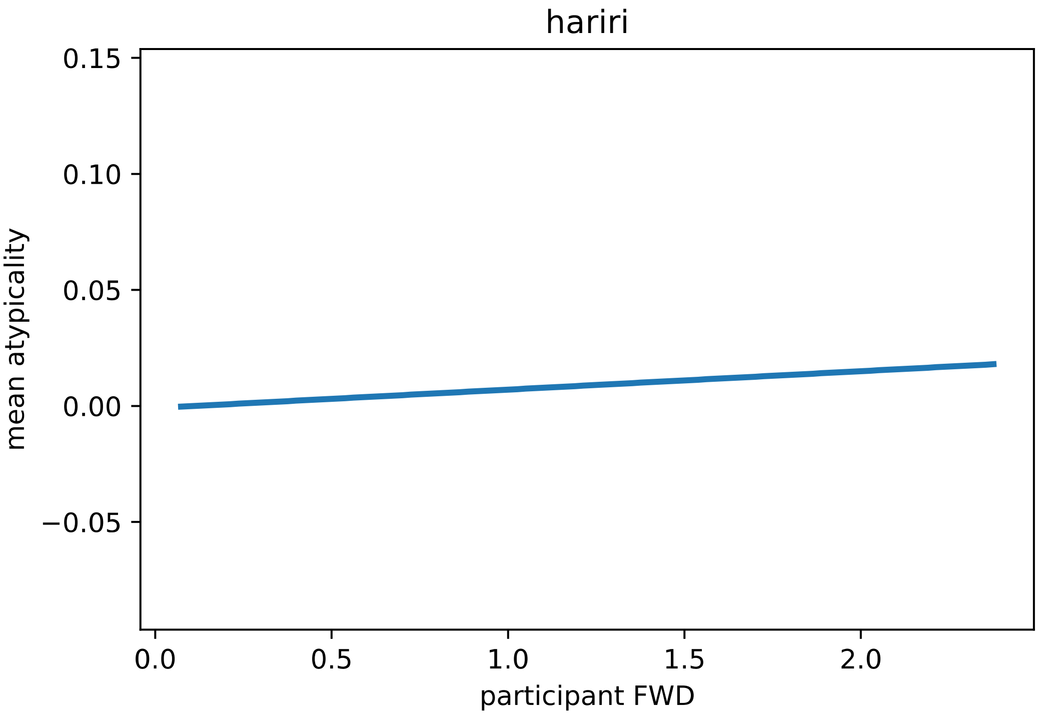

Figure supp. 3A.

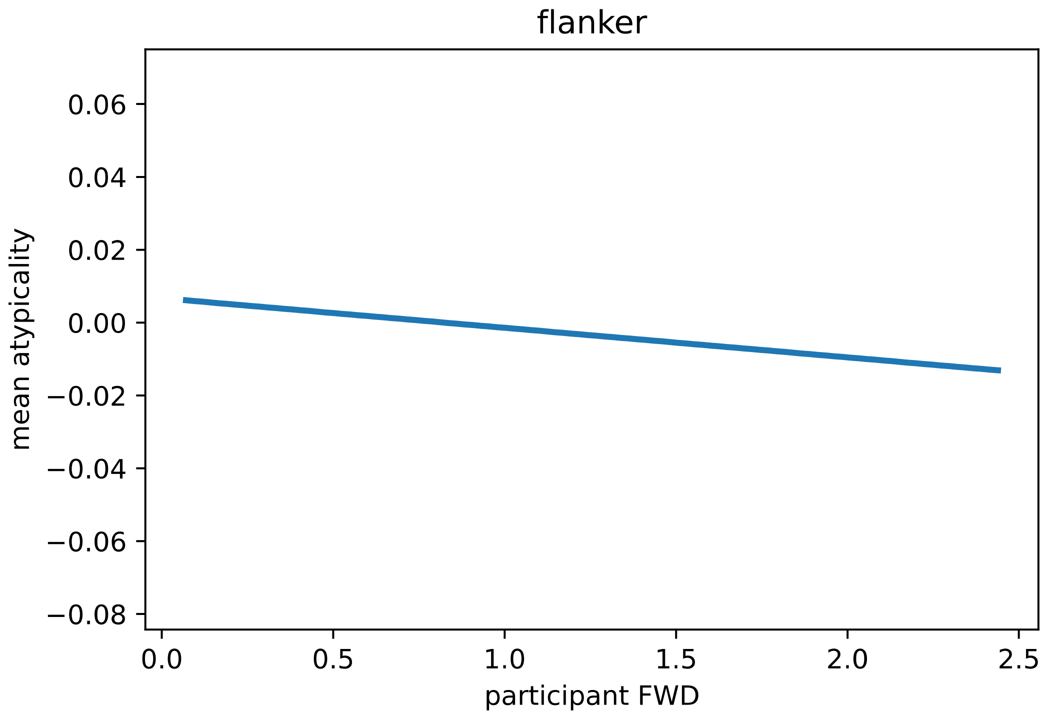

Figure supp 3B.

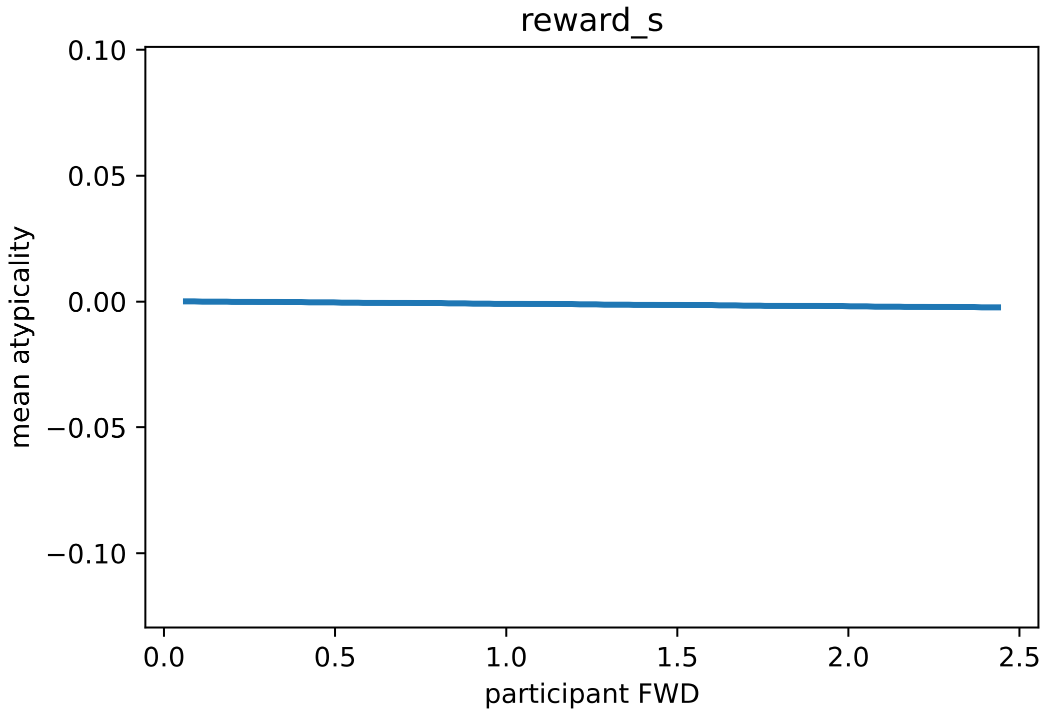

Figure supp 3C.

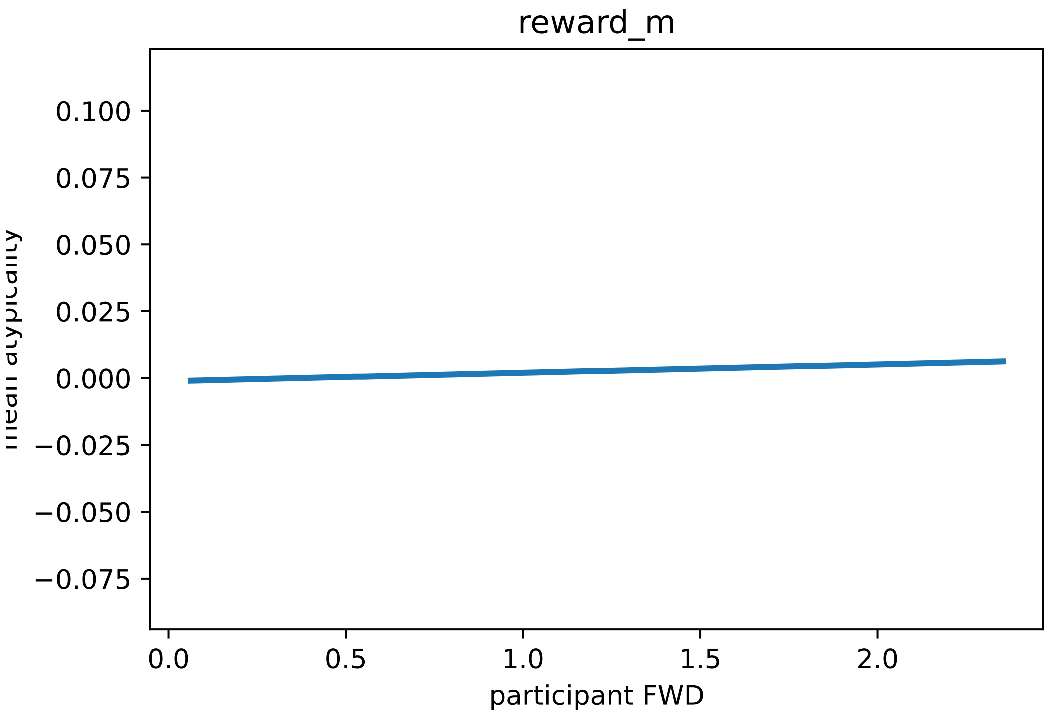

Figure supp 3D.

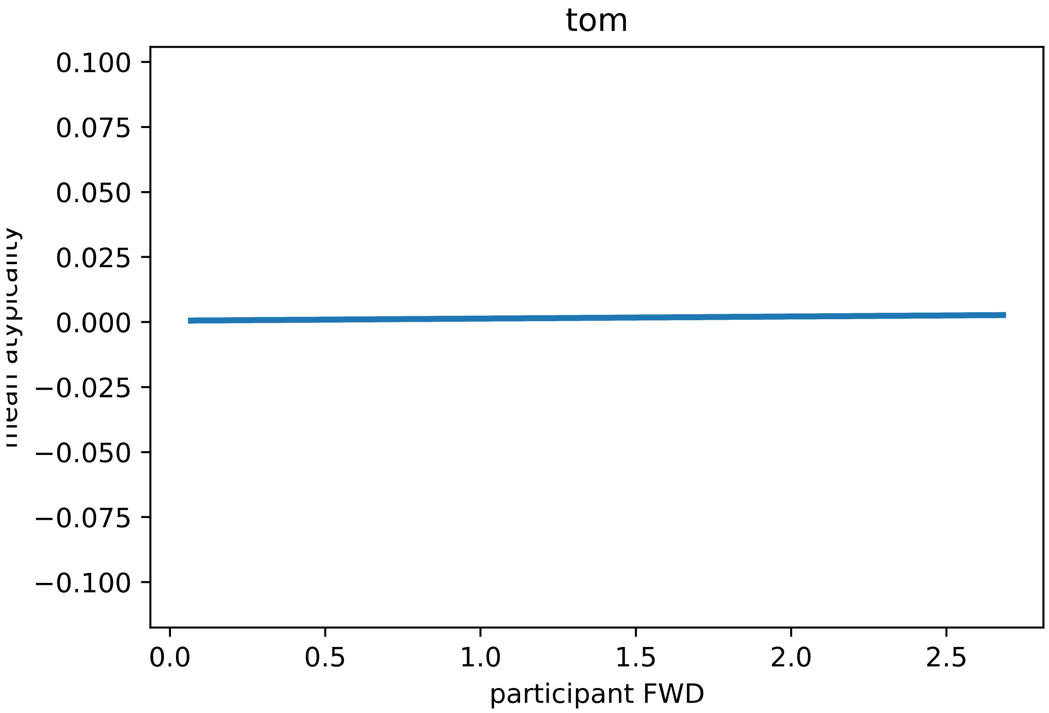

Figure supp 3E.

Figures 4A-E

Figures with the mean FC matrices for autism per task, with and without site correction. Each figure also includes a figure of a matrix that shows the difference between the matrices with and without site correction. Note that the scale on the difference matrix is ten times smaller than on the original matrices

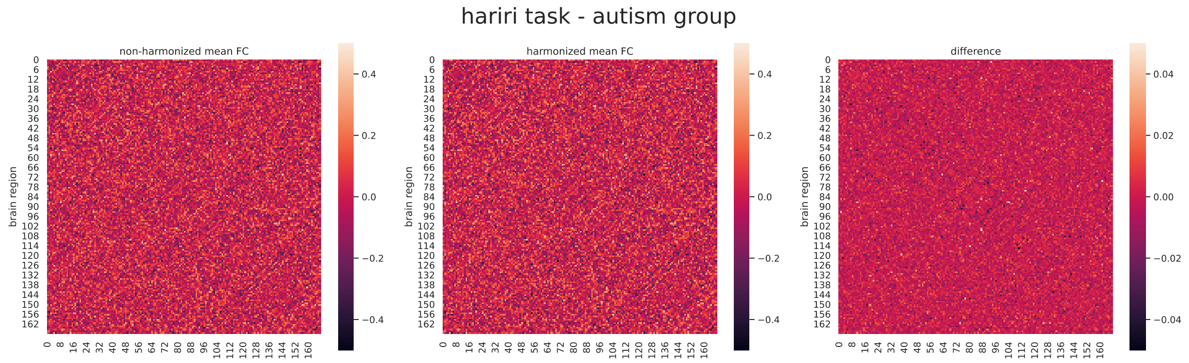

Figure supp 4A

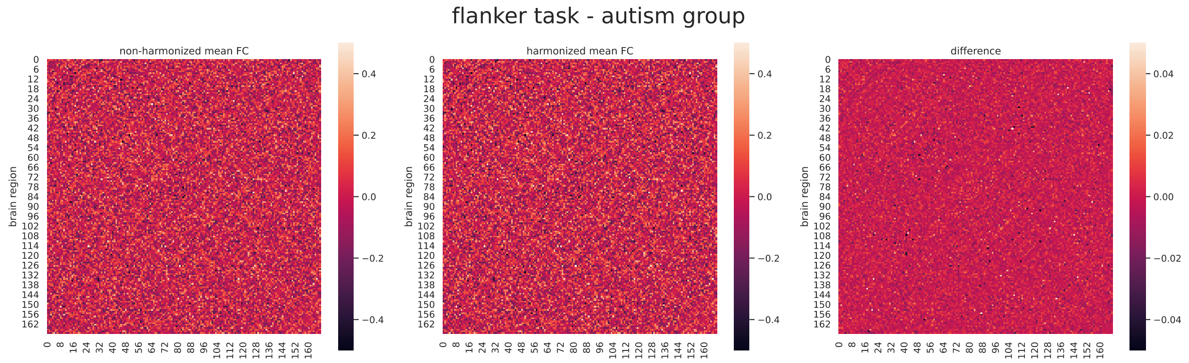

Figure supp 4B

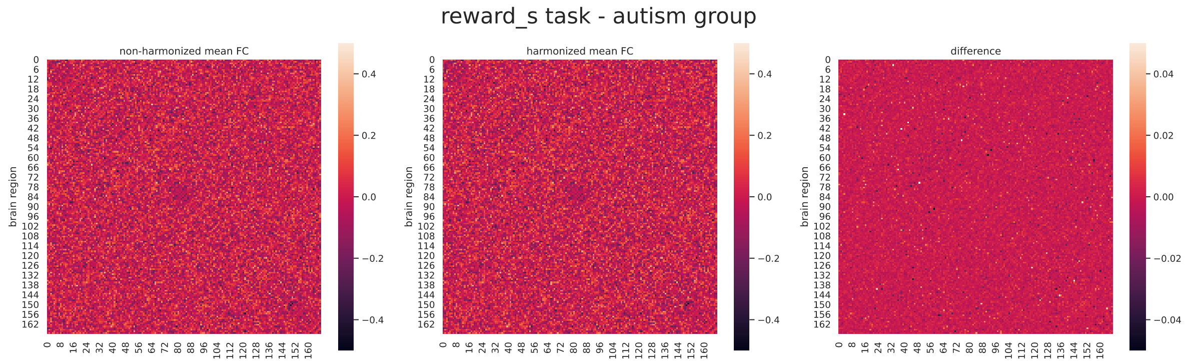

Figure supp 4C

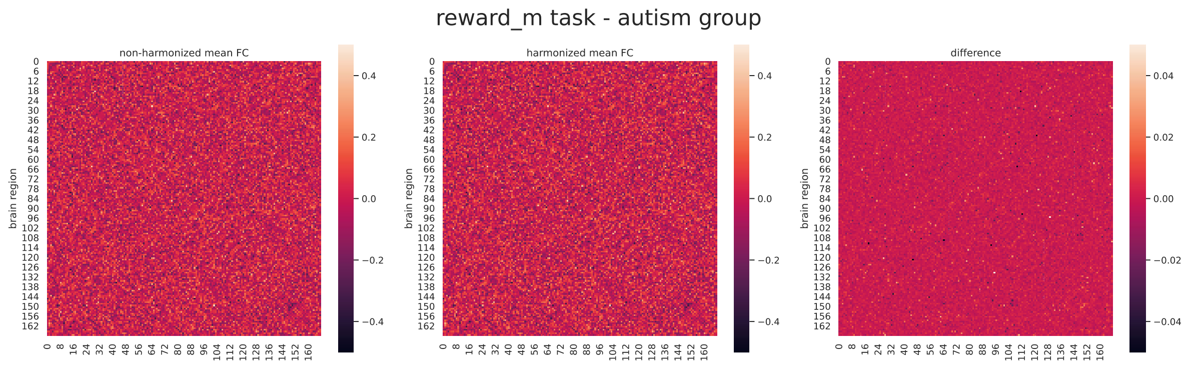

Figure supp 4D

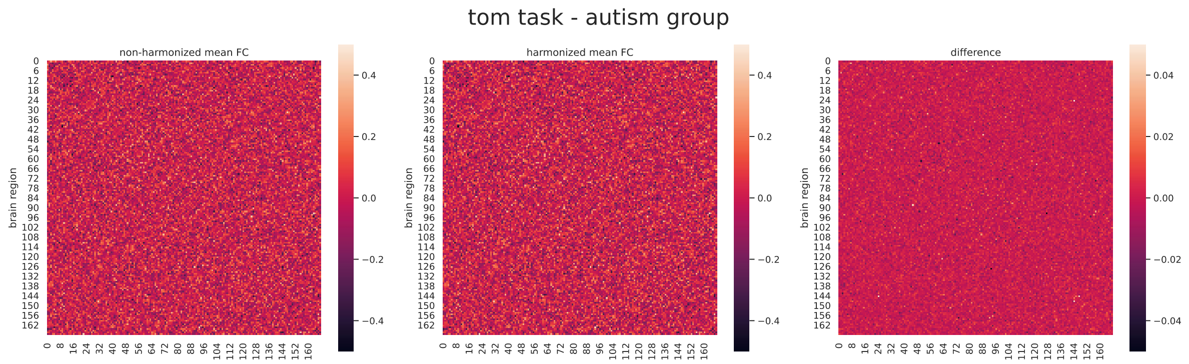

Figure 4E
